# Association Between Feeling Upon Awakening and Use of Information Technology Devices in Japanese Children

**DOI:** 10.2188/jea.JE20110019

**Published:** 2012-01-05

**Authors:** Yusuke Kondo, Tsuyoshi Tanabe, Mikiko Kobayashi-Miura, Hiroki Amano, Natsu Yamaguchi, Masanori Kamura, Yasuyuki Fujita

**Affiliations:** 1 Department of Public Health, Shimane University Faculty of Medicine, Shimane, Japan; 2 Izumo First Junior High School Zone Sukoyaka Task Force, Shimane, Japan

**Keywords:** Shimane prefecture, information technology devices, feeling upon awakening, school health

## Abstract

**Background:**

The objective of this study was to clarify the relationship between feeling upon awakening (FA) and time spent using information technology (IT) devices by children in kindergartens, elementary schools, and junior high schools in Shimane, Japan.

**Methods:**

In October 2008, a self-report survey was distributed to 2075 children in kindergartens (*n* = 261), elementary schools (*n* = 1162), and junior high schools (*n* = 652) in Shimane, Japan. The questionnaire gathered data on sex, school year, feeling upon awakening, and time spent using IT devices after school (television, videos on television, video games, personal computers, and cellular phones). After adjusting for sex and school year, data were analyzed by multivariate logistic regression analysis to calculate odds ratios (ORs) and 95% confidence intervals (CIs).

**Results:**

A total of 2030 children completed this survey (response rate, 97.8%). Negative FA was associated with watching television more than 2 hours/day (OR = 1.51, 95% CI = 1.23–1.85), playing video games more than 30 minutes/day (1.50, 1.20–1.87), and using personal computers more than 30 minutes/day (1.35, 1.04–1.75).

**Conclusions:**

Time spent using IT devices affected the FA of children in kindergarten through junior high school. We propose the development of guidelines regarding the appropriate amount of time this population should spend using IT devices.

## INTRODUCTION

It is important to clarify the relationship between the health of children and their use of information technology (IT) devices. Prolonged use of IT devices has been reported to be related to fatigue, sleep habits, and childhood obesity. Van den Bulck^1^ reported that use of IT devices led to significantly later bedtimes. Studies in Japan and other countries have reported relationships between obesity, fatigue, and prolonged use of IT devices.^2^^–^^7^

Clarification of the relationships among sleep habits, growth, and health is important for primary prevention efforts among children. Gaina et al conducted a survey of Japanese junior high school students and reported that insufficient sleep caused daytime sleepiness.^8^ These results led the authors to propose better sleep habits, increased physical activity, and restriction of television viewing time. The Izumo First Junior High School Zone Sukoyaka Task Force distributed a lifestyle questionnaire to kindergarten, elementary, and junior high school students in this school zone, which is located in Shimane Prefecture. We conducted a cross-sectional study to clarify the association between IT device use and feeling upon awakening (FA) using the epidemiologic information obtained by the Task Force. FA was used as an index of sleep satisfaction.

## METHODS

### Subjects

The lifestyle questionnaire was a self-administered survey conducted by the Task Force in October 2008, and was distributed to 2075 potential participants in Shimane Prefecture. In this report, “preschool” refers to any educational institution attended before entering elementary school. In Japan, preschools mainly include kindergartens and nursery schools. Kindergartens care for children from 3 to 6 years of age for about 4 hours per day. Nursery schools care for toddlers to children aged 6 years for 8 to 12 hours per day. The sample included 261, 1162, and 652 students, respectively, from 4 kindergartens, 3 elementary schools, and 1 junior high school.

### Lifestyle questionnaire

The questionnaire gathered data on sex, school year, the preschool circumstances of first graders, FA on weekdays, time of awakening, bedtime, sleep duration, and time spent using IT devices (television, video games, personal computers, and cellular phones) on weekdays after school. The questionnaire distributed to kindergarteners and children in first through third grades contained 21 items; all others received a 23-item questionnaire.

The item inquiring about sleep duration was intended only for kindergarteners, fourth through sixth graders, and junior high school students. The preschool circumstances of first graders were coded as follows: 1, kindergarten; 2, nursery school; and 3, other. FA was coded as 1, good; 2, not too bad; 3, not too good; and 4, bad. Time of awakening was classified into the following categories: 1, <6:30 AM; 2, ≥6:30 AM to <7:00 AM; 3, ≥7:00 AM to <7:30 AM; and 4, ≥7:30 AM. Bedtime was classified as 1, <9:00 PM; 2, ≥9:00 PM to <10:00 PM; 3, ≥10:00 to <11:00 PM; 4, ≥11:00 to <12:00 PM; and 5, ≥12:00 PM. Sleep duration was coded as 1, ≥9 h; 2, 8 to 9 h; 3, 7 to 8 h; and 4, <7 h. Time spent watching television daily was categorized as 1, very little; 2, <1 h; 3, 1 to 2 h; 4, 2 to 3 h; 5, 3 to 4 h; and 6, ≥4 h. Use of video games daily was coded as 1, non-player; 2, <0.5 h; 3, 0.5 to 1 h; 4, 1 to 2 h; 5, 2 to 3 h; 6, 3 to 4 h; and 7, ≥4 h. Time spent using cellular phones daily was coded as 1, do not use; 2, <0.5 h; 3, 0.5 to 1 h; 4, 1 to 2 h; 5, 2 to 3 h; 6, 3 to 4 h; and 7, ≥4 h. Time spent using personal computers daily was categorized as 1, do not use; 2, <0.5 h; 3, 0.5 to 1 h; 4, 1 to 2 h; 5, 2 to 3 h; 6, 3 to 4 h; 7, ≥4 h. Elementary and junior high school students completed the questionnaires by themselves. Questionnaires for kindergarteners were completed by their guardians. Teachers distributed questionnaires to elementary and junior high school students, and these were completed and collected immediately.

### Statistical analyses

We conducted the chi-square test to assess the relationship between FA and other factors (time of awakening, bedtime, sleep duration, and time spent using IT devices). After adjusting for sex and school year, data were analyzed by multivariate logistic regression analysis to calculate odds ratios (ORs) and 95% confidence intervals (CIs).

Answers were dichotomized into 2 categories. FA was divided into “good” (good, not too bad) and “bad” (not too good, bad). Time of awakening was classified as before 6:30 AM and 6:30 AM or later. Bedtime was dichotomized as before 10:00 PM and 10:00 PM or later. Sleep duration was divided into <8 h and ≥8 h. Time spent watching television was categorized as <2 h and ≥2 h. Time spent using video games was dichotomized into <0.5 h and ≥0.5 h. Time spent using cellular phones was grouped into <0.5 h and ≥0.5 h. Time spent using personal computers was dichotomized into <0.5 h and ≥0.5 h. Statistical analyses incorporated FA, time of awakening, bedtime, sleep duration, and time spent using IT devices. Children with missing data for FA, time of awakening, bedtime, sleep duration, or time spent using IT devices were excluded from each analysis. Statistical analyses were performed with SAS version 9.2 (SAS Institute, Cary, NC, USA).

Collinearity between independent factors was evaluated by calculating the variance inflation factor (VIF) using the PROC REG procedure. The values were 1.06 for time of awakening, 1.72 for bedtime, 1.57 for sleep duration, 1.08 for television viewing, 1.24 for playing video games, 1.12 for cellular phone use, 1.05 for personal computer use, 1.21 for sex, and 1.65 for age. Because none of the VIFs exceeded 2, we concluded that collinearity was not a concern.

To determine the cutoff point for IT use time, the OR for bad FA, after adjusting for sex and school year, was calculated for several possible cutoff points, including <1 h or ≥1 h, <2 h or ≥2 h, <3 h or ≥3 h, and <4 h or ≥4 h for time spent watching television, and <0.5 h or ≥0.5 h, <1 h or ≥1 >h, <2 h or ≥2 h, <3 h or ≥3 h, and <4 h or ≥4 h for time spent playing video games, computer use, and cellular phone use. The proportion of children with bad FA significantly increased when categorized as <2 h or ≥2 for time spent watching television, and as >0.5 h or ≥0.5 h for time spent playing video games and using computers. Bad FA did not increase significantly with any categorization for time spent for using cellular phones. Thus, in subsequent analysis, we used cutoff points of <2 h or ≥2 h for time spent watching television and <0.5 h or ≥0.5 h for time spent playing video games, using computers, and using cellular phones.

Shimane University’s Institutional Committee on Ethics approved this study.

## RESULTS

### Subjects

A total of 2030 children responded to the questionnaire; however, 45 did not reply because they were absent when the questionnaires were collected. The response rate was 97.8%. Children with data missing for sex or school year were excluded from the analyses. Ultimately, data from 2028 children were analyzed.

### Lifestyle questionnaire

Table 1 shows the number of respondents by sex, type of school, and school year. There was no significant difference in the numbers of boys and girls (999 and 1029, respectively). The sample included 247 children from kindergarten, 1149 from elementary school, and 632 from junior high school. The minimum number of students in any elementary and junior high school was 184 and the maximum was 226. Each kindergarten included 69 to 97 children, which represented approximately 50% of elementary and junior high school students per school year. Eighty-nine children had entered elementary school from kindergartens, 110 from nursery schools, and 1 from another type of institution. Thus, 44.7% of first-grade students had progressed from kindergarten.

**Table 1.** Respondents by educational stage

Educational level	Boys	Girls	Total
	*n* (%)	*n* (%)	*n* (%)
Kindergarten			
First year	35 (43.2)	46 (56.8)	81 (100)
Second year	42 (60.9)	27 (39.1)	69 (100)
Third year	46 (47.4)	51 (52.6)	97 (100)
Total	123 (49.8)	124 (50.2)	247 (100)
Elementary school			
First year	89 (44.5)	111 (55.5)	200 (100)
Second year	101 (53.7)	87 (46.3)	188 (100)
Third year	102 (52.0)	94 (48.0)	196 (100)
Fourth year	96 (51.1)	92 (48.9)	188 (100)
Fifth year	81 (42.0)	112 (58.0)	193 (100)
Sixth year	91 (49.5)	93 (50.5)	184 (100)
Total	560 (48.7)	589 (51.3)	1149 (100)
Junior high school			
First year	115 (50.9)	111 (49.1)	226 (100)
Second year	108 (50.0)	108 (50.0)	216 (100)
Third year	93 (49.0)	97 (51.1)	190 (100)
Total	316 (50.0)	316 (50.0)	632 (100)

Total	999 (49.3)	1029 (50.7)	2028 (100)

Table 2 shows sleep–wake patterns and media use among children in kindergartens, elementary schools, and junior high schools, based on our data on FA, time of awakening, bedtime, sleep duration, and time spent using IT devices. The proportion of those who played video games for more than half an hour per day was significantly greater for boys than for girls (*P* < 0.01), while the proportion of those who used cellular phones for more than half an hour per day was significantly greater for girls than for boys (*P* < 0.01). We found no significant differences between boys and girls in FA, time of awakening, bedtime, sleep duration, or time spent watching television or using personal computers.

**Table 2.** Sleep-wake pattern and media use by sex among children in kindergarten, elementary school, and junior high school

	Boys	Girls	*P*-value^c^
	*n* (%)	*n* (%)	
Feeling upon awakening			
Good	722 (72.3)	757 (73.6)	0.51
Bad	276 (27.7)	271 (26.4)	
Total	998 (100.0)	1028 (100.0)	
Time of awakening			
<6:30	303 (30.4)	319 (31.2)	0.71
≥6:30	693 (69.6)	704 (68.8)	
Total	996 (100.0)	1023 (100.0)	
Bedtime			
<22:00	551 (55.3)	557 (54.1)	0.61
≥22:00	446 (44.7)	472 (45.9)	
Total	997 (100.0)	1029 (100.0)	
Sleep duration^a^			
<8 h	426 (60.3)	427 (57.9)	0.37
≥8 h	281 (39.8)	310 (42.1)	
Total	707 (100.0)	737 (100.0)	
Television			
<2 h	613 (61.5)	631 (61.4)	0.96
≥2 h	384 (38.5)	397 (38.6)	
Total	997 (100.0)	1028 (100.0)	
Video games			
<30 min	523 (52.5)	840 (82.1)	<0.01
≥30 min	473 (47.5)	183 (17.9)	
Total	996 (100.0)	1023 (100.0)	
Cellular phone^b^			
<30 min	831 (94.9)	791 (87.6)	<0.01
≥30 min	45 (5.1)	112 (12.4)	
Total	876 (100.0)	903 (100.0)	
Personal computer^b^			
<30 min	702 (80.2)	742 (82.3)	0.27
≥30 min	173 (19.8)	160 (17.7)	
Total	875 (100.0)	902 (100.0)	

^a^This question was intended only for children in kindergarten, fourth through sixth grade, or junior high school.

^b^This question was intended only for elementary and junior high school students.

^c^The chi-square test was performed.

Table 3 summarizes the numbers of respondents, sleep–wake patterns, and media use among kindergarten, elementary, and junior high school students. There were significant differences among these groups with respect to sleep–wake pattern and media use, including FA, time of awakening, bedtime, sleep duration, and time spent using IT devices (TV, video games, personal computers, and cellular phones) (*P* < 0.01).

**Table 3.** Sleep-wake pattern and media use by educational status among children in kindergarten, elementary school, and junior high school

	Kindergarten	Elementary school	Junior high school	*P*-value^b^
	*n* (%)	*n* (%)	*n* (%)	
Feeling upon awakening				
good	213 (86.2)	877 (76.5)	389 (61.6)	
bad	34 (13.8)	270 (23.5)	243 (38.5)	
total	247	1147	632	<0.01
Time of awakening				
<6:30	40 (16.2)	414 (36.3)	168 (26.6)	
≥6:30	207 (83.8)	726 (63.7)	464 (73.4)	
total	247	1140	632	<0.01
Bedtime				
<22:00	232 (93.9)	816 (71.1)	60 (9.5)	
≥22:00	15 (6.1)	331 (28.9)	572 (90.5)	
total	247	1147	632	<0.01
Sleep duration				
≥8 h	244 (98.8)	432 (76.5)	177 (28.0)	
<8 h	3 (1.2)	133 (23.5)	455 (72.0)	
total	247	565	632	<0.01
Television				
<2 h	166 (67.2)	786 (68.6)	292 (46.2)	
≥2 h	81 (32.8)	360 (31.4)	340 (53.8)	
total	247	1146	632	<0.01
Video games				
<30 min	221 (89.5)	731 (64.1)	411 (65.0)	
≥30 min	26 (10.5)	409 (35.9)	221 (35.0)	
total	247	1140	632	<0.01
Personal computer^a^				
<30 min	—	1007 (88.0)	437 (69.2)	
≥30 min	—	138 (12.1)	195 (30.9)	
total	—	1145	632	<0.01
Cellular phone^a^				
<30 min	—	1096 (95.6)	526 (83.2)	
≥30 min	—	51 (4.5)	106 (16.8)	
total	—	1147	632	<0.01

^a^This question was intended for elementary and junior high school students.

^b^The chi-square test was performed.

The Figure shows the frequency of negative FA according to sex, school, and year in school. We found higher percentages in junior high school than in elementary school students, and higher percentages in elementary school than in kindergarten students. However, the percentages did not increase linearly for every school year in each school and kindergarten.

**Figure.**
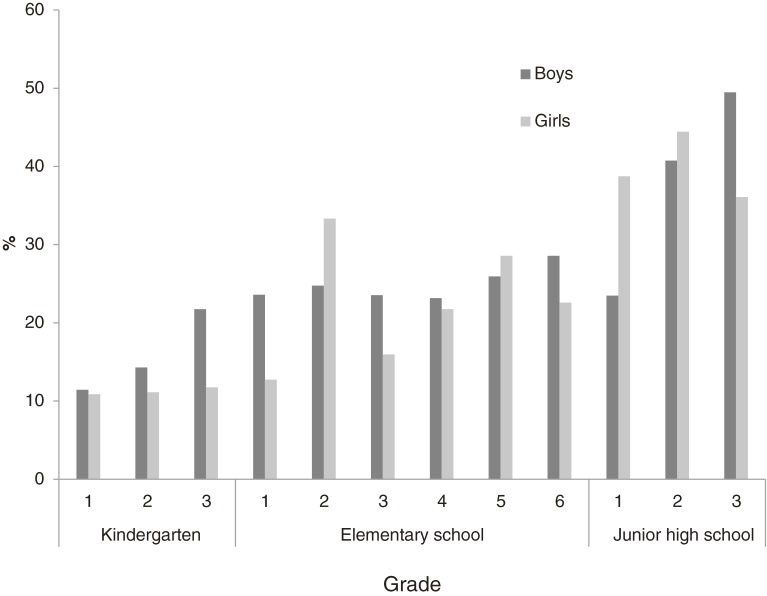
Frequency of negative feeling upon awakening (FA) according to sex, school, and year in school

Table 4 shows the ORs and 95% CIs for FA by sex among children in kindergartens, elementary schools, and junior high schools, according to the results of multivariate logistic regression analysis. The analysis included variables associated with FA, ie, time of awakening, bedtime, sleep duration, and time spent using IT devices. The overall adjusted ORs for negative FA were 1.96 (95% CI: 1.54–2.48) for awakening after 6:30 AM, 2.49 (1.46–3.25) for a bedtime of 10:00 PM or later, 1.92 (1.46–2.53) for a sleep duration less than 8 h, 1.51 (1.23–1.85) for watching television for 2 h or more, 1.50 (1.20–1.87) for playing video games for 0.5 h or more, and 1.35 (1.04–1.74) for using a personal computer for more than 0.5 h. The prevalence of bad FA increased as a function of awakening after 6:30 AM, going to bed after 10:00 PM, sleeping less than 8 hours per night, watching television more than 2 h, and using video games or personal computers for more than half an hour.

**Table 4.** ORs and 95% CIs for negative feeling upon awakening by sex among children in kindergarten, elementary school, and junior high school

	Boys		Girls		Total	
		
	OR^a^	95% CI	OR^a^	95% CI	OR^b^	95% CI
Time of awakening						
<6:30	1		1		1	
≥6:30	2.32	1.64–3.29	1.7	1.23–2.36	1.96	1.54–2.48
Bedtime						
<22:00	1		1		1	
≥22:00	2.61	1.81–3.74	2.33	1.56–3.47	2.49	1.91–3.25
Sleep duration						
≥8 h	1		1		1	
<8 h	1.69	1.16–2.47	2.21	1.47–3.32	1.92	1.46–2.53
Television						
<2 h	1		1		1	
≥2 h	1.23	0.92–1.65	1.84	1.37–2.47	1.51	1.23–1.85
Video games						
<30 min	1		1		1	
≥30 min	1.28	0.96–1.71	1.94	1.38–2.74	1.5	1.2–1.87
Personal computer						
<30 min	1		1		1	
≥30 min	1.49	1.05–2.13	1.2	0.82–1.75	1.35	1.04–1.75
Cellular phone						
<30 min	1		1		1	
≥30 min	1.34	0.71–2.51	1.05	0.67–1.63	1.15	0.8–1.65

^a^OR and 95% CI were calculated by multivariate logistic regression analysis adjusted for school year.

^b^OR and 95% CI were calculated by multivariate logistic regression analysis adjusted for sex and school year.

Relationships between FA and bedtime, sleep duration, and time of awakening were evident in both boys and girls. Among girls, watching television and playing video games were significantly associated with negative FA. An association between FA and time spent using personal computers was observed only in boys. There was no significant association between cellular phone use and FA.

Table 5 shows the ORs and 95% CIs for FA by type of school according to multivariate logistic regression analysis of FA, time of awakening, bedtime, sleep duration, and time spent using IT devices. We found a significant relationship between FA and time of awakening, bedtime, sleep duration, and time spent using video games among students in elementary and junior high schools. Elementary school students who watched more than 2 hours of television and spent more than half an hour using personal computers were significantly more likely to feel negative FA.

**Table 5.** ORs and 95% CIs for negative feeling upon awakening by educational status among children in kindergarten, elementary school, and junior high school

	Kindergarten		Elementary school		Junior high school		Total	
			
	OR^a^	95% CI	OR^a^	95% CI	OR^a^	95% CI	OR^a^	95% CI
Time of awakening								
<6:30	1		1		1		1	
≥6:30	2.25	0.65–7.79	2.59	1.86–3.6	1.54	1.05–2.26	1.96	1.54–2.48
Bedtime								
<22:00	1		1		1		1	
≥22:00	1.56	0.41–5.89	2.59	1.9–3.53	3.07	1.51–6.24	2.49	1.91–3.25
Sleep duration								
≥8 h			1		1		1	
<8 h			1.92	1.26–2.93	1.93	1.3–2.87	1.92	1.46–2.53
Television								
<2 h	1		1		1		1	
≥2 h	1.27	0.6–2.69	1.79	1.34–2.4	1.35	0.98–1.87	1.51	1.23–1.85
Video games								
<30 min	1		1		1		1	
≥30 min	1.32	0.45–3.9	1.72	1.28–2.31	1.48	1.01–2.17	1.5	1.2–1.87
Personal computer								
<30 min			1		1		1	
≥30 min			1.63	1.11–2.41	1.11	0.78–1.57	1.35	1.04–1.75
Cellular phone								
<30 min			1		1		1	
≥30 min			1.52	0.83–2.8	0.86	0.54–1.35	1.15	0.8–1.65

^a^OR and 95% CI were calculated by multivariate logistic regression analysis adjusted for sex and school year.

We analyzed only kindergarteners to represent preschoolers. We compared the influence of various factors on how children felt upon awakening and determined whether there were differences between elementary school students who had graduated from kindergarten and those who graduated from nursery school. Table 6 shows the ORs and 95% CIs for FA among elementary school children who had graduated from kindergarten or nursery school. Relationships of FA with time of awakening and bedtime were evident in kindergarten and nursery school graduates. The association between FA and time spent playing video games was observed only in kindergarten graduates. FA was associated with sleep duration and television viewing only among nursery school graduates. Personal computer use and cellular phone use were not significantly associated with FA.

**Table 6.** ORs and 95% CIs for negative feeling upon awakening among elementary school students who graduated from kindergarten and nursery school

	Kindergarten		Nursery school	
	
	OR^a^	95% CI	OR^a^	95% CI
Time of awakening				
<6:30	1		1	
≥6:30	2.55	1.54–4.20	2.36	1.51–3.69
Bedtime				
<22:00	1		1	
≥22:00	3.09	1.87–5.11	2.28	1.50–3.46
Sleep duration				
≥8 h	1		1	
<8 h	1.74	0.85–3.56	2.00	1.14–3.52
Television				
<2 h	1		1	
≥2 h	1.45	0.91–2.32	1.92	1.29–2.86
Video games				
<30 min	1		1	
≥30 min	2.09	1.31–3.33	1.33	0.89–1.98
Personal computer				
<30 min	1		1	
≥30 min	1.79	0.97–3.30	1.53	0.90–2.62
Cellular phone				
<30 min	1		1	
≥30 min	1.28	0.46–3.59	1.77	0.81–3.87

^a^OR and 95% CI were calculated by multivariate logistic regression analysis adjusted for sex and school year.

## DISCUSSION

The objective of this study was to clarify the relationship between FA and the use of IT devices among children in kindergartens, elementary schools, and junior high schools in the task force area. We found that children who spent more than 2 hours watching television, more than half an hour playing video games, and more than half an hour using personal computers were more likely to report bad FA. The present findings show a significant relationship between time spent using IT devices and bad FA. Additionally, those who awakened after 6:30 AM, went to bed after 10:00 PM, and slept less than 8 hours at night were more likely to report bad FA.

Because sleeping time, dietary habits, and physical activity are affected by area of residence, it is important to note that the data used in this survey were collected from the same school zone. To clarify the relationship of time spent using IT devices with health and lifestyle, it is important to keep the region constant. We used the same questionnaires and the same manuals for each school to increase the reliability of the data.

Our results revealed that watching TV more than 2 hours per day was significantly correlated with bad FA. In their analysis of the sleep habits of 2546 Belgian students in their first or fourth year of secondary school, Van den Bulck et al found an association between TV viewing time and sleep problems.^1^ They noted that weekly time spent watching television correlated significantly with less weekday sleep and higher levels of tiredness. Johnson et al found that children who watched television more than 3 hours per day during adolescence were at a significantly higher risk of frequent sleep problems by early adulthood.^9^ They also noted that adolescents who reduced their television viewing from 1 hour or more per day to less than 1 hour per day significantly reduced their risk of subsequent sleep problems.

Other reports have shown that watching television for more than 2 hours per day causes sleep problems, which agrees with the present findings.^10^^–^^12^ A study of 19 299 elementary school children in China showed that watching television for more than 2 hours per day on weekends was a risk factor for sleep disorders such as bedtime resistance, sleep onset delay, sleep anxiety, and night awakening.^10^ Similar findings were reported by Owens et al, who demonstrated that 2 or more hours of weekday television viewing caused sleep problems among students in kindergarten through fourth grade.^11^^,^^12^ The American Academy of Pediatrics has recommended that youth should not watch more than 1 to 2 hours of television per day.^13^ Our findings provide support for this recommendation.

The present results strongly indicate that time spent using computers and playing video games significantly affects FA. The association between IT time and sleep problems has been documented. For example, Punamaki et al examined sex and age differences relative to the intensity of IT use.^14^ They surveyed 7292 Finnish children aged 12, 14, 16, and 18 years and examined the possible mediating role of sleeping habits and tiredness upon awakening with regard to the association with IT use and perceived health status. They showed that intensive computer use by boys and intensive cellular phone use by girls were associated with health risks. Van den Bulck et al showed that children who spent more time playing computer games and using the Internet went to bed later, awakened later, spent less time in bed, and reported higher levels of tiredness.^1^ Another study investigated 1143 Japanese school children aged 6 to 11 years and found that playing television games for more than 1 hour per day was linked to sleep deprivation and symptoms of daytime sleepiness.^15^ Choi et al examined the association of Internet overuse with excessive daytime sleepiness^16^ and showed that the prevalences of insomnia, witnessed snoring, apnea, teeth grinding, and nightmares were highest in Internet addicts, average in possible addicts, and lowest in non-addicts. By showing that more than half an hour playing video games and more than half an hour using personal computers influence sleep problems, our results suggest an acceptable range for IT use.

Our study revealed a noteworthy sex difference: watching TV for more than 2 hours per day was significantly associated with bad FA only among girls. Gaina et al demonstrated that longer TV viewing was followed by increased sleepiness risk in Japanese junior high school students, especially girls.^8^ They showed that boys exhibited significant increases in sleepiness after 3 hours, in contrast to girls whose risk increased after only 1 hour. In addition, longer viewing (>4 hours) was associated with a higher risk of sleepiness in girls. These results could be due to the higher prevalence of general sleep problems in Japanese girls.^17^ Moreover, a higher prevalence of excessive daytime sleepiness among females has been reported in Brazil^18^ and in Korea.^19^ Other studies have reported no difference^20^ or a higher prevalence among males.^21^

This study focused on kindergarten, elementary school, and junior high school students aged 3 to 15 years. This population differs from those of other studies that investigated much more limited student populations.^1^^,^^8^^,^^11^^,^^12^^,^^22^^,^^23^ The FA ratios of the present participants can be arranged in descending order as junior high school > elementary school > kindergarten. These results partially confirm those of other studies of Japanese students, which showed that elementary schoolchildren seemed to sleep for a sufficient period of time, whereas students attending junior or senior high schools did not.^24^

The present study revealed that only elementary school students showed an association between FA and the use of 3 IT devices (TVs, personal computers, and video games), implying a stronger etiologic link between IT device use and sleep problems among elementary school students than among kindergarteners and junior high school students. Several studies of elementary school students have reported similar observations.^10^^,^^22^^,^^23^ For example, a study of elementary school students in China showed that watching TV for more than 2 hours per day caused sleep problems.^10^

The present study had limitations that warrant discussion. Because this was a cross-sectional survey, the causal relationship between IT use and sleep problems cannot be determined. This makes it necessary for us to continue our research as a longitudinal study.

Our questionnaire did not include all possible factors that might adversely affect sleep. In addition to IT use, sleep problems can be caused by demographic factors (eg, age, sex, and ethnicity),^25^^,^^26^ socioeconomic status (eg, family income, family structure, and parental educational levels),^25^^,^^26^ sleep environment (eg, intrusive bedroom noise),^27^ children’s chronic health problems (eg, obesity, chronic respiratory conditions, and chronic pain),^28^^,^^29^ school schedules (eg, earlier start times, more time required for homework),^30^^,^^31^ bedtime hygiene (eg, having drinks with caffeine after 6:00 PM, exciting activities before bedtime, and having irregular bedtime),^32^ and parental sleep habits (eg, shorter sleep duration).^33^ Li et al showed that IT use, school schedule, and parental sleep habits had a greater effect than sleep environment and chronic health problems on children’s sleep problems.^25^ Several studies showed that a TV in a child’s room significantly modified sleep–wake patterns and was the most significant predictor of overall sleep disturbance and bedtime resistance.^1^^,^^10^^,^^12^ The content of television/computer programs may also correlate with sleep problems, because excessively violent or stimulating programming might inhibit relaxation, resulting in difficulty falling asleep.^34^ Therefore, we will include questions concerning these factors in future questionnaires.

In Japan, elementary and junior high school attendance is compulsory. In the area of our present study, there were few private elementary or junior high schools. Most preschool children attend kindergarten or nursery school. In the present investigation, we studied only kindergarteners. In the study area, most mothers of nursery school children work, whereas the mothers of kindergarteners are primarily housewives. Therefore, sleep habits and media contact times might be different between kindergarteners and nursery schooler: and would be expected to influence sleep habits and time spent using media among elementary school students. Furthermore, we showed that the correlations between FA and each factor differed among elementary school students who had graduated from kindergarten and nursery school. An analysis of nursery school students will be included in our future studies because not including them here may have introduced bias into the results.

In conclusion, we have identified factors concerning media use that influence FA awakening and have proposed time limits for watching television, playing video games, and using personal computers. We will continue our research in a longitudinal study to clarify more precisely the relationships between media use and sleep problems.

## APPENDIX

Questionnaire on media use

1. Sex
① male ② female
2. Type of school
① kindergarten ② elementary school ③ junior high school
3. Grade
① First ② Second ③ Third ④ Fourth ⑤ Fifth ⑥ Sixth
4. What kind of preschool did you graduate from?
① kindergarten ② nursery ③ other
5. Do you wake up with a good feeling in the morning?
(feeling upon awakening, FA)
① good ② not too bad ③ not too good ④ bad
6. What time do you wake up in the morning?
①1. <6:30 AM ② ≥6:30 AM to <7:00 AM ③ ≥7:00 AM to <7:30 AM ④ ≥7:30 AM
7. What time do you go to bed in the evening?
① <9:00 PM ② ≥9:00 PM to <10:00 PM ③ ≥10:00 PM to <11:00 PM ④ ≥11:00 PM to <12:00 PM ⑤ ≥12:00 PM
8. How long do you sleep? (not including grades 1–3 in elementary school)
① ≥9 hours ② ≥8 hours to <9 hours ③ ≥7 hours to <8 hours ④ <7 hours
9. How long do you watch TV (including video) in a day?
① very little ② <1 hour ③ ≥1 hour to <2 hours ④ ≥2 hours to <3 hours ⑤ ≥3 hours to <4 hours ⑥ ≥4 hours (  hours)
10. How long do you play videogames in a day?
① non-player ② <30 min. ③ ≥30 min. to <1 hour ④ ≥1 hour to <2 hours ⑤ ≥2 hours to <3 hours ⑥ ≥3 hours to <4 hours ⑦ ≥4 hours (  hours)
11. How long do you use a cellular phone in a day?
① do not use ② <30 min. ③ ≥30 min. to <1 hour ④ ≥1 hour to <2 hours ⑤ ≥2 hours to <3 hours ⑥ ≥3 hours to <4 hours ⑦ ≥4 hours (  hours)
12. How long do you use a personal computer in a day?
① do not use ② <30 min. ③ ≥30 min. to <1 hour ④ ≥1 hour to <2 hours ⑤ ≥2 hours to <3 hours ⑥ ≥3 hours to <4 hours ⑦ ≥4 hours (  hours)
